# Neurometabolomic impacts of wood smoke and protective benefits of anti-aging therapeutics in aged female C57BL/6J mice

**DOI:** 10.1186/s12989-025-00639-4

**Published:** 2025-09-01

**Authors:** David Scieszka, Jonathan Hulse, Haiwei Gu, Amanda Barkley-Levenson, Ed Barr, Marcus Garcia, Jessica G. Begay, Guy Herbert, Mark McCormick, Jonathan Brigman, Andrew Ottens, Barry Bleske, Kiran Bhaskar, Matthew J. Campen

**Affiliations:** 1 Department of Pharmaceutical Sciences, 1 University of New Mexico College of Pharmacy, MSC09 5360, Albuquerque, NM 87131-0001 USA; 2 Department of Molecular Genetics and Microbiology, Department of Neurology, School of Medicine, University of New Mexico Albuquerque, Albuquerque, NM 87131 USA; 3 College of Health Solutions, Arizona State University, Phoenix, AZ 85004 USA; 4 Department of Biochemistry and Molecular Biology, School of Medicine, University of New Mexico Health Sciences Center, Albuquerque, NM 87131 USA; 5 Department of Neurosciences, University of New Mexico School of Medicine, Albuquerque, NM 87131 USA; 6 Department of Anatomy and Neurobiology, Virginia Commonwealth University, Richmond, VA 23298 USA; 7 Department of Pharmacy Practice and Administrative Sciences, University of New Mexico College of Pharmacy, Albuquerque, NM 87131 USA

**Keywords:** Particulate matter, Wildfire smoke, Metabolomics, Brain, Depression, Wildland fire, Wood smoke, Anti-aging

## Abstract

**Background:**

Wildland fires in the United States have increased in frequency and scale over the past 30 years exposing millions of people to hazardous air pollutants. Among others, aging individuals are particularly vulnerable to the effects of wildfire smoke. In this study, we assessed the neurobiological impacts of wood smoke (WS) on aged mice and the potential of anti-aging therapeutics to mitigate these impacts.

**Methods:**

Female C57BL/6 J mice, aged 18 months, were divided into 10 groups and exposed to either filtered air (FA; 5 groups) or biomass derived WS (5 groups) for 4 h/day, every other day, for 14 days (7 exposures total) with an average particulate matter (PM) concentration of 448 µg/m^3^ per exposure. One FA control group and one WS exposed group were euthanized 24 h after the last exposure. The remaining 8 groups (4 FA and 4 WS exposed) were treated with either vehicle control, resveratrol and nicotinamide mononucleotide (RNMN), dasatinib and quercetin (DQ), or both RNMN and DQ (RNDQ) for 10 weeks.

**Results:**

A significant reduction in NAD + within the prefrontal cortex was observed following the 14-day exposure to WS along with a reduction in serotonin. Serotonin reductions were observed up to 10 weeks post-exposure and co-occurred with neuroinflammation and behavioral alterations, including increased immobility in a forced swim test. RNMN conferred the greatest mitigating effect after WS exposure, while RNDQ treatment resulted in an upregulation of markers associated with aging in the brain. While the metabolic shift in the PFC following WS exposure was relatively modest, mice exposed to FA and vehicle control (10 weeks of natural aging) exhibited the greatest metabolic shift, including perturbed nicotinamide metabolism.

**Conclusion:**

Taken together, these findings highlight that subacute (14-day) exposure to WS results in persistent neurometabolomic and behavioral alterations in an aged mouse model and that intervention with RNMN may be a useful strategy to mitigate the adverse neurological outcomes observed. Further studies are needed to assess the specific impact of either resveratrol or NMN in isolation and to fully elucidate age-specific, as well as sex- and species-determinant, WS exposure response pathways.

**Supplementary Information:**

The online version contains supplementary material available at 10.1186/s12989-025-00639-4.

## Introduction

In the United States over the past 30 years there has been a documented increase in the frequency and scale of wildfires, which release airborne particulate matter [1] and gaseous pollutants. The total acres burned per year in the United States have roughly doubled over the past two decades [61]. Particulate Matter (PM)_2.5_ from wildfires is now negating positive air quality trends in numerous regions, especially in the western United States [1]. Degraded air quality can impact populations up to thousands of kilometers from the fire source [2–5]. Recent epidemiological [14, 15] and preclinical animal [16, 17] studies document the potential of wildfire smoke exposure to cause adverse neurological outcomes. In humans, Alzheimer's disease related dementias (ADRD) [6, 7], suicide [6, 8], depression [6, 9], psychosis [6, 10], and other neurological outcomes have been associated with exposure to particulate matter (PM) irrespective of sources [11–13], and specifically from wildfire smoke [14]. Older individuals are especially vulnerable due to age-related decline in respiratory and cardiovascular function [63, 64]. With wildfire activity attributed largely to global climate change and increased aridity—trends that are projected to intensify—there is a critical need to identify strategies to mitigate WS-associated risks to public health.

Pharmacological interventions hold promise for conferring cognitive benefits and cardioprotective effects, which we conjecture could offset wildfire smoke-induced neurological and cardiovascular conditions. Wildfire smoke has been associated with pulmonary senescence fates [18] as well as adverse neurological outcomes arising from inflammation [16, 19]. Senescence is a proinflammatory state of cell cycle arrest that has been proposed as a root cause of aging [20–23], resulting in systemic inflammation through the release of senescence-associated secretory phenotype molecules into the circulation [24]. To counteract this release, senolytic agents such as dasatinib and quercetin target senescent cell anti-apoptotic pathways, promoting apoptosis and cell clearance [25].

Resveratrol is a natural polyphenol with demonstrated anti-inflammatory [26, 27], anti-cancer [27, 28], neuroprotective [26, 27], and cardioprotective properties [27, 29, 30], the effects of which are mediated through activation of sirtuin 1 and other nicotinamide adenine dinucleotide (NAD^+^)-dependent enzymes. Nicotinamide mononucleotide (NMN) is a NAD^+^ precursor [31], whose supplementation has been shown as beneficial for cellular NAD^+^ abundance, longevity [32], cognition [33], ADRD [34], and depression [35], among others [36]. A growing number of studies indicate that resveratrol [37, 38] and nicotinamide mononucleotide (NMN) [39, 40, 40] have antioxidant properties, while healthspan benefits have been reported from supplementation with a combination of dasatinib + quercetin (DQ) [41–43]. Only one recent study has investigated the combined effects of resveratrol with NMN (RNMN) [44], and no studies have combined all four agents (RNDQ). Therefore, we examined the potential of RNMN, DQ, and RNDQ to mitigate WS-induced neurological impairments in aged mice.

For the current study, metabolomic alterations in the prefrontal cortex (PFC) were queried based on its known role in cognitive disorders and neurodegenerative diseases, both of which have been reported in population level studies of wildfire smoke [14, 38]. We then followed up on discoveries from neurometabolomics by evaluating specific behavioral and neuroimmune outcomes of subacute WS exposure. The objectives of the study were to 1) identify potential persistent neurobiological effects of subacute WS after cessation of exposure, 2) determine whether WS exposure alters the trajectory of aging across a period of decline (from 18 to 21 months of age in a mouse model), and 3) to evaluate the potential of various anti-aging interventions to diminish WS-induced neurometabolomic impacts.

## Results

### Exposure conditions: particulate matter concentration and size distribution

To examine neurometabolomic effects of wildfire smoke, whole-body inhalation exposures to WS or filtered air (FA) were conducted for 4 h (h) every other day for 14 days (7 total exposures). Female 18-month-old mice were exposed to an average PM concentration of 448 µg/m^3^ for each of the 4 h exposures (Supplemental Fig. 1), with an average daily exposure concentration (in line with U.S. Environmental Protection Agency regulatory methods) of 37 µg/m^3^ for the 14-day period to mimic fluctuating concentrations seen in earlier studies of naturally-occurring wildfire PM [16, 17] and best mirroring variances in human exposures that occur routinely during summer months [62]. Carbon monoxide and oxides of nitrogen were also monitored to reflect the gaseous component of biomass combustion emissions, and these averaged 3.2 ppm and 4 ppb, respectively (Supplemental Fig. 1). Carbon monoxide and oxides of nitrogen species were well below U.S. Environmental Protection Agency National Ambient Air Quality Standard levels. (It is worth noting that the U.S. Environmental Protection Agency routinely excludes wildfire-contributed PM and gases from ambient PM measures for regulatory purposes). PM size distribution was acquired without mice in the exposure chamber, and distribution was measured over a single 2 h run. Particle size distribution largely fell within PM_1_ (median range = 0.138–0.145 μm) with less than 1% of particles (by number) above PM_2.5_. WS exposures did not impact body weight trends in any of the pharmaceutical interventions, compared to FA (Supplemental Fig. 2).

### Prefrontal cortex untargeted metabolomic effects of biomass smoke

**Fig. 1:**
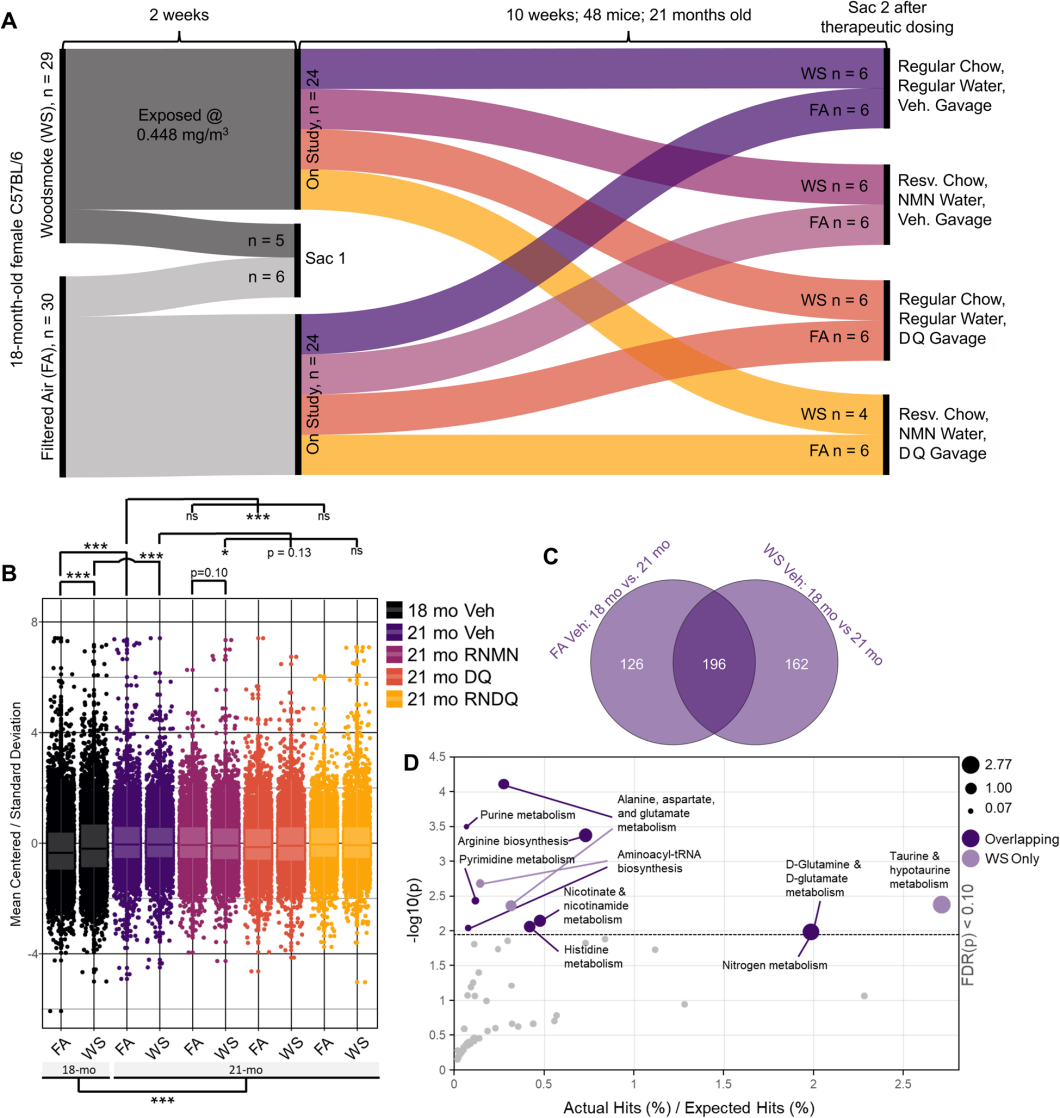
Schematic of experimental design and initial difference testing and metabolomic aging verification. **A** Experimental design schematic. 18-month-old female C57BL/6 J mice were exposed to filtered air (FA) or woodsmoke (WS) at an average concentration of 448 µg/m.^3^ every other day for 2 weeks (7 total exposure groups; *N* = 6/group). 1 day post exposures, one cohort of FA and WS (18 mo) were euthanized (sac). The remaining mice were divided into the following intervention groups: vehicle control (Veh), resveratrol plus nicotinamide mononucleotide (RNNM), dasatinib + quercitin (DQ) or a combination of both (RNDQ); mice were treated for 10 weeks. At the end of the intervention, each cohort (21 mo) was euthanized for metabolomic sequencing. **B** Linear mixed effects regression modeling for the entire metabolomic dataset. **C** Venn diagram of statistically significant metabolites (Student’s t-tests). **D** Significant pathways affected by metabolites that overlapped (dark purple) and those that were exclusive to WS (light purple). KEGG pathways for *Mus musculus*. ns = not significant, *p ≤ 0.05, **p ≤ 0.01, *p ≤ 0.001

To assess the long-term persistence of neurometabolomic effects and potential protection from resveratrol, NMN, and senolytics, after the last exposure, two groups of mice were euthanized (*n* = 6 FA and *n* = 5 WS-exposed mice) while the remaining FA and WS-exposed mice were equally divided into 1 of the 4 intervention groups (vehicle control, RNNM, DQ or RNDQ) for 10 weeks after exposures (Fig. 1A). The intervention protocols were as follows: 1. Vehicle control intervention: standard chow, deionized (DI) water, gavage with vehicle; 2. RNMN intervention: resveratrol milled into standard chow, NMN added in DI water, gavage with vehicle; 3. DQ intervention: standard chow, DI water, DQ given via gavage; 4. RNDQ intervention: resveratrol milled into standard chow, NMN added in DI water, DQ given via gavage.

Of the 40 total 18-month-old mice exposed to WS, only one died during the 14-day exposure period, while no FA controls died. In the subsequent 10 weeks of pharmacological treatment, another two of the WS mice (out of 34) died before study completion, both in the RNDQ treatment arm. The cause of death was unclear. No FA mice died prematurely.

We observed significant metabolomic differences between 18-month-old FA and WS-exposed animals in the PFC (Fig. 1B). In addition, the natural course of aging significantly altered PFC metabolites as indicated through the comparisons of 18-month-old vehicle control animals to 21-month-old vehicle control animals (observed for both FA and WS groups), as well as the grouped comparison of 18-month-old FA and WS to 21-month-old FA and WS (Fig. 1B and also illustrated by the principal component analysis in Supplemental Fig. 4). The RNMN intervention group showed a trend toward a distinct PFC metabolomic signature compared to other 21-month-old FA vs WS exposure conditions, which was significantly different from the exposure-matched WS 21-month-old vehicle controls. There were no differences observed in the animals from the FA RNMN group compared to those from the FA vehicle control group. However, analysis of the PFCs from the FA DQ intervention group revealed a significantly different metabolomic profile compared to age-matched FA vehicle controls. These findings indicate that, in mice not exposed to WS, DQ had the strongest effect on the overall PFC metabolic profile (in terms of numbers of significantly altered metabolites), while RNMN had the strongest effect on WS-exposed mice.

#### Aging influence on prefrontal cortex metabolites

To interrogate the natural aging effect observed between animals 18 months of age and 21 months of age, we compared the significantly altered PFC metabolites for both the FA and WS-exposed groups (Fig. 1C). We observed 196 shared metabolites between the groups. The number of unique metabolites were 126 and 162 for FA and WS, respectively. The cluster of shared metabolites was functionally associated with aging-related metabolomic pathways that were unperturbed by WS exposure. We extracted the significant, shared metabolites and conducted pathway analysis to identify enriched pathways. After the false discover rate (FDR) correction (cut-off *p* < 0.1), the enriched pathways included alanine, aspartate, and glutamate metabolism; purine biosynthesis; arginine biosynthesis; pyrimidine metabolism; and nicotinate and nicotinamide metabolism (Fig. 1D, Supplemental Table 1). The affected pathway of nicotinate and nicotinamide metabolism confirms the accuracy of pathway analysis employed, based on abundant literature showing that aging is associated with declines in NAD^+^ reserves and biosynthesis [45–55]. We then took the uniquely identified WS metabolites and queried them for pathway alterations. Previous work from our lab has shown that wildfire exposures decrease neuroprotective taurine [16], which we confirmed here as one of the main affected pathways: taurine and hypotaurine metabolism. Taken together, these results indicate that the effect of aging over this 10-week period in mice had a greater impact on the metabolic profile than the effects of our subacute WS exposure. Additionally, aging had a greater impact on the PFC of mice regardless of pharmaceutical interventions. Additionally, the altered metabolic profile observed following WS exposures persisted up to 10 weeks after exposure cessation. The intervention that showed the greatest effect in the PFC’s of FA and WS were DQ and RNMN, respectively.

#### The effects of wood smoke exposure and pharmaceutical interventions on prefrontal cortex metabolites

**Fig. 2:**
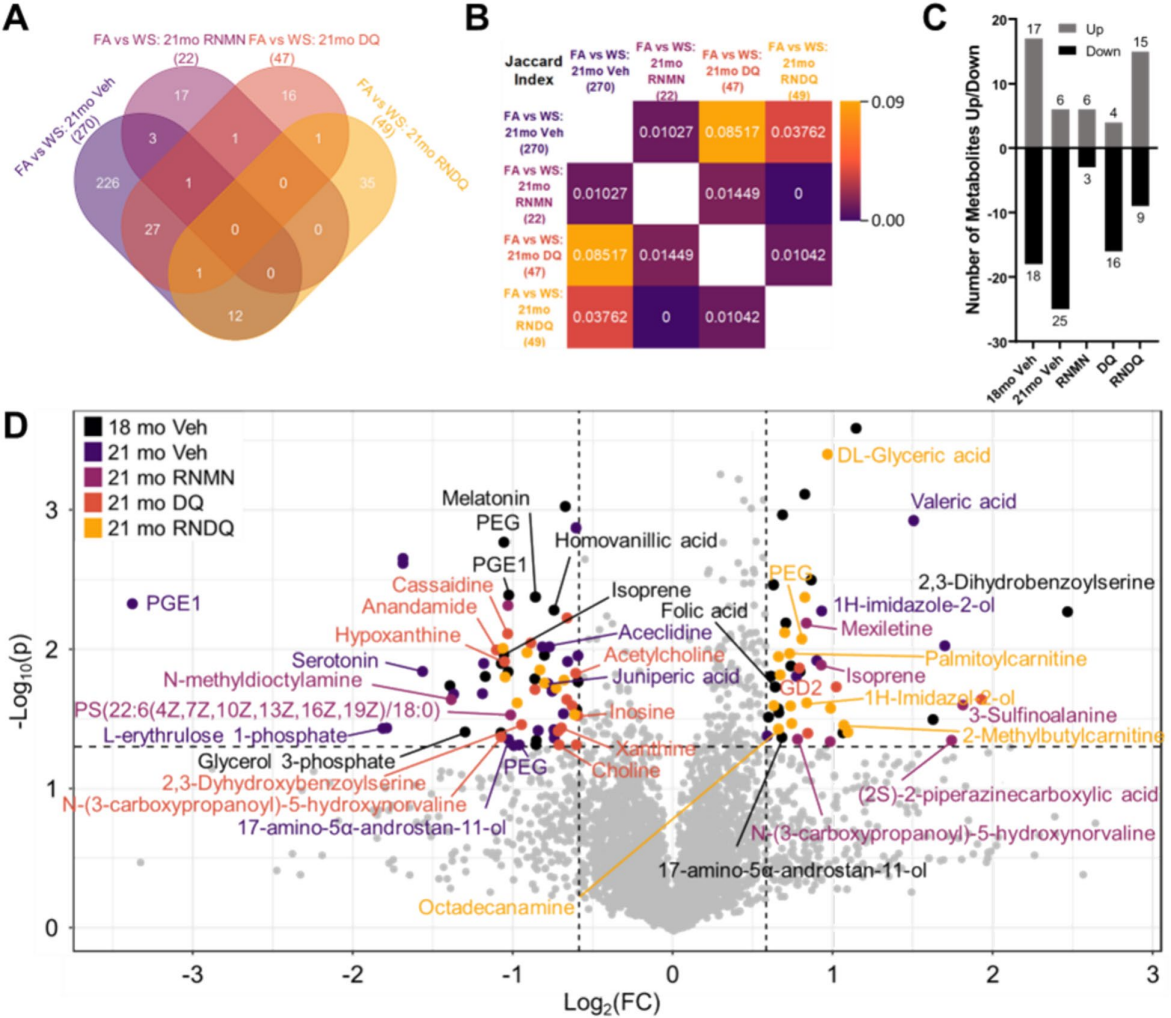
Drug correlations and overlay of significantly altered metabolites. **A** Venn diagram of statistically significant metabolites (Student’s t-tests). **B** For each group, Jaccard indices were calculated (**C**) Significant volcano-plot-calculated metabolites up/down per condition comparing FA versus WS. **D** Overlay of untargeted and NAD + metabolomic panels. In order from top to bottom, Black: young, 18mo-old mice. Dark purple: natural course of aging for 10 weeks after WS exposure without drug intervention. Light purple: resveratrol and NMN. Orange: dasatinib and quercetin (DQ). Yellow: RNMN + DQ (RNDQ)

To distinguish alterations to the metabolic profile by subacute exposure to WS from the natural effects of aging we compared the PFCs of the FA-exposed mice in the vehicle control intervention group to those of WS-exposed mice in the vehicle intervention group at the 21 month timepoint (Fig. 2). The efficacy of the anti-aging interventions in mitigating the neurometabolomic impact of the WS exposure was assessed by comparing the profiles of the WS-exposed mice from the intervention groups to the age matched FA vehicle control group (Fig. 2). Pathway analysis revealed 270 uniquely identified metabolites in the PFCs of the vehicle control group and 22, 47, and 49 uniquely identified metabolites in the PFCs of mice in the RNMN, DQ, and RNDQ intervention groups, respectively. The weakest overlap was observed between vehicle control group and the RNMN intervention group (3 unique + 1 shared with DQ), followed by the vehicle group and the RNDQ group [13]. The strongest overlap was seen between the vehicle control and the DQ intervention group (29; Fig. 2B). However, these overlaps were relatively small compared to the overall outcomes of FA exposure vs WS exposure following 10 weeks of recovery without intervention (270). These findings indicate that intervention with DQ had the smallest effect on the perturbed PFC profile following WS exposure, and that the combination of RNMN resulted in the greatest benefit. We also employed a Jaccard calculation to remove weighted bias based on the total number of significantly altered metabolites per condition. Regardless of methodology employed, we see the strongest overlap between vehicle and DQ treatment (J_index_: 0.08517), a medium overlap between vehicle and RNDQ (0.0376), and the weakest overlap between vehicle and RNMN treatment (0.0103; Fig. 2B). These data indicate a minor reduction in the effectiveness of the DQ and RNDQ intervention after WS exposure, which prompted a thorough investigation into individual metabolite contributions.

To this end, volcano plots were compiled for intervention-age-matched exposure groups (FA vs WS). The number of significantly up and downregulated metabolites revealed several patterns (Fig. 2C). The largest number of significantly *decreased* metabolites were observed in FA mice treated with vehicle compared to WS-exposed mice treated with vehicle. This was true for both age groups (18 mo: 18 metabolites; 21 mo: 25 metabolites). In contrast, 18-month-old vehicle-treated mice compared to RNDQ-treated mice had the greatest number of significantly *increased* metabolites (18 mo: 17; RNDQ:15; Fig. 2C). Out of all the pharmaceutical interventions employed, RNDQ treatment resulted in the largest number of significantly altered metabolites, observed in the FA vs WS-exposed mice PFCs [24], while treatment with RNMN resulted in the lowest number of significantly altered metabolites [9]. Together, our findings show that 10-weeks of natural recovery was inefficient at resolving WS-induced alterations to the metabolic profile of the PFC, and that treatment with RNMN most effectively resolved WS changes, while RNDQ elicited the greatest differential drug response between FA and WS. Taken together, these data illustrate the differences seen at each level of examination (with and without corrections). Without FDR correction, metabolomic analysis revealed broad profiles that highlighted an aging effect (Figs. 1C; 2 A, B). With FDR correction, pathway analyses, lmer, and volcano plots identified the metabolites underlying the observed shifts in the metabolic profiles (Figs. 1B, D; 2D).

Overall, we observed a greater shift in the metabolic profile of the 18-month-old WS-exposed vehicle-treated animals than we expected from a subacute exposure (Fig. 2D). Interestingly, treatment with RNDQ resulted in upregulation of two metabolites known to exist within the aging murine brain (DL-glyceric acid [56] and octadecylamine [57]). This finding indicates that treatment with RNDQ after WS exposure may exacerbate aging in the PFC. In contrast, our data suggest that treatment with RNMN may aid in resolving metabolic perturbations caused by subacute WS exposure, a finding not observed in animals treated with DQ alone or RNDQ (Fig. 2C, D).

### Targeted PFC pathway metabolomics

**Fig. 3:**
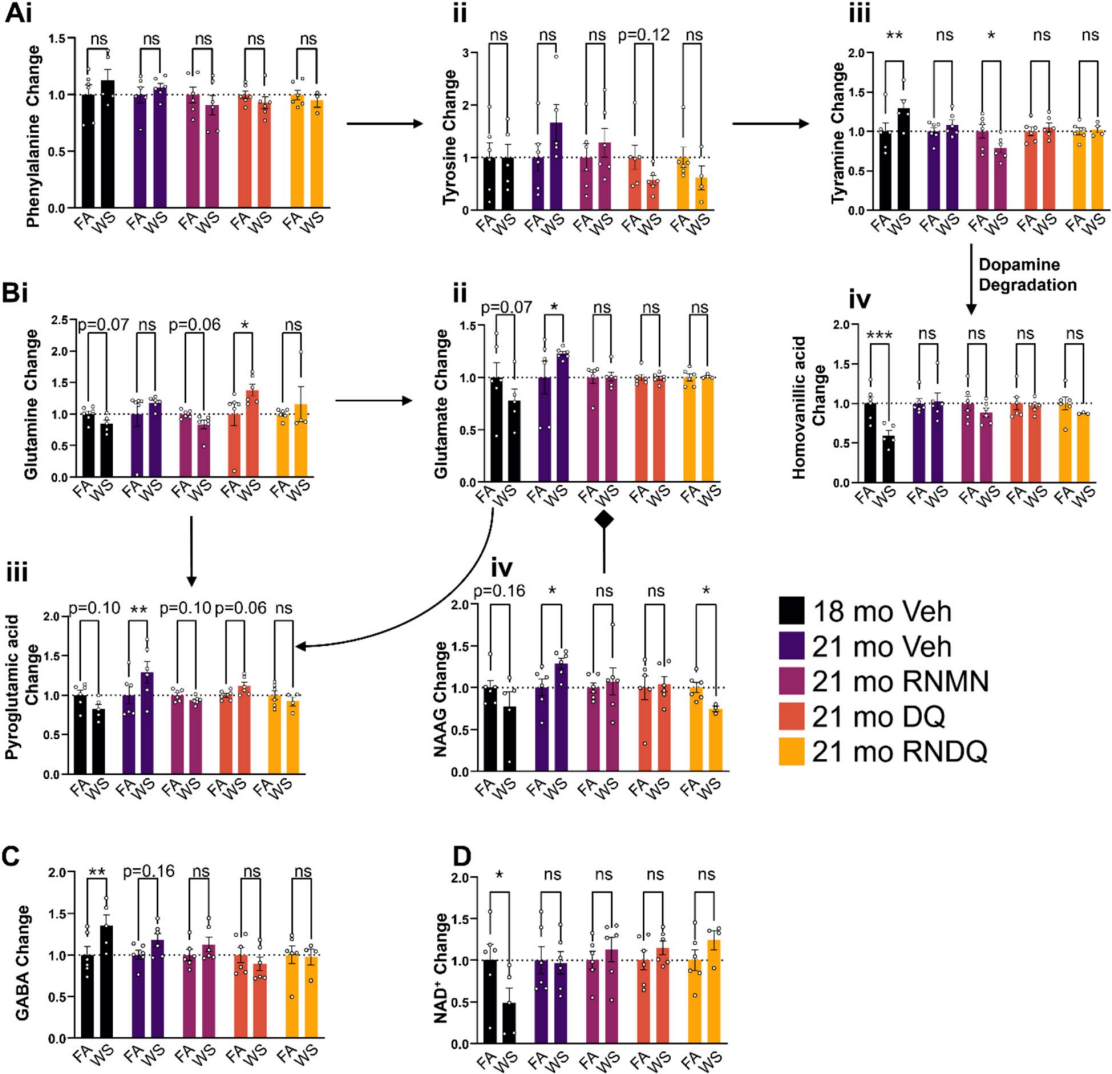
De novo neuro-metabolite investigations. **A**i-iv) Metabolites detected within the dopamine synthesis and degradation pathway. **B** i-iv) Metabolites detected in the tripart glutamine, glutamate, and pyroglutamic pathway along with NAAG, which facilitates the release of glutamate. **C** GABA D) NAD +. Asterisks denote significant difference from control (*p < 0.05; **p < 0.01; *P* < 0.001)

A targeted panel of metabolites was used to specifically examine serotonin and dopamine pathways in the PFC, as well as N-acetylaspartylglutamic acid (N-acetylaspartylglutamate or NAAG), GABA and NAD^+^. Untargeted metabolomic analysis revealed changes in dopamine pathway metabolites including phenylalanine, tyrosine, tyramine, and homovanillic acid (Fig. 3 A i-iv), although dopamine itself was not detected in this analysis. At 1-day post-exposure, 18-month-old vehicle-treated mice exhibited elevated tyramine (Fig. 3A iii) and reduced homovanillic acid (Fig. 3A iv), which are the upstream and downstream metabolites of dopamine, respectively. The tripartite glutamine, glutamate, pyroglutamate pathway (Fig. 3B i-iii) illustrates an increase in glutamate at the 21-month timepoint. NAAG facilitates the release of glutamate (Fig. 3B iv) and was thus examined as a potential cause for the elevated glutamate observed in 21-month-old vehicle-treated mice. From these 4 panels, the downward trending glutamate observed in the 18-month-old vehicle-treated mice cannot be explained by changes in related metabolites in this tripartite pathway. However, these data suggest that NAAG is partially responsible for the increased glutamate and pyroglutamic acid in 21-month-old vehicle-treated mice. Additionally, GABA was elevated 1-day post exposure in the 18-month-old vehicle-treated animals, and we observed trending increases 10 weeks later in the 21-month-old vehicle-treated animals (Fig. 3C). NAD^+^ was decreased immediately after WS exposure, indicating the potential for accelerated neurological aging (Fig. 3D). Taken together, these data reveal long-term effects of WS exposure on serotonin, glutamate, NAAG, and GABA (10 weeks), many of which are able to be resolved through the combinations of RNMN, DQ, or RNDQ.

**Fig. 4:**
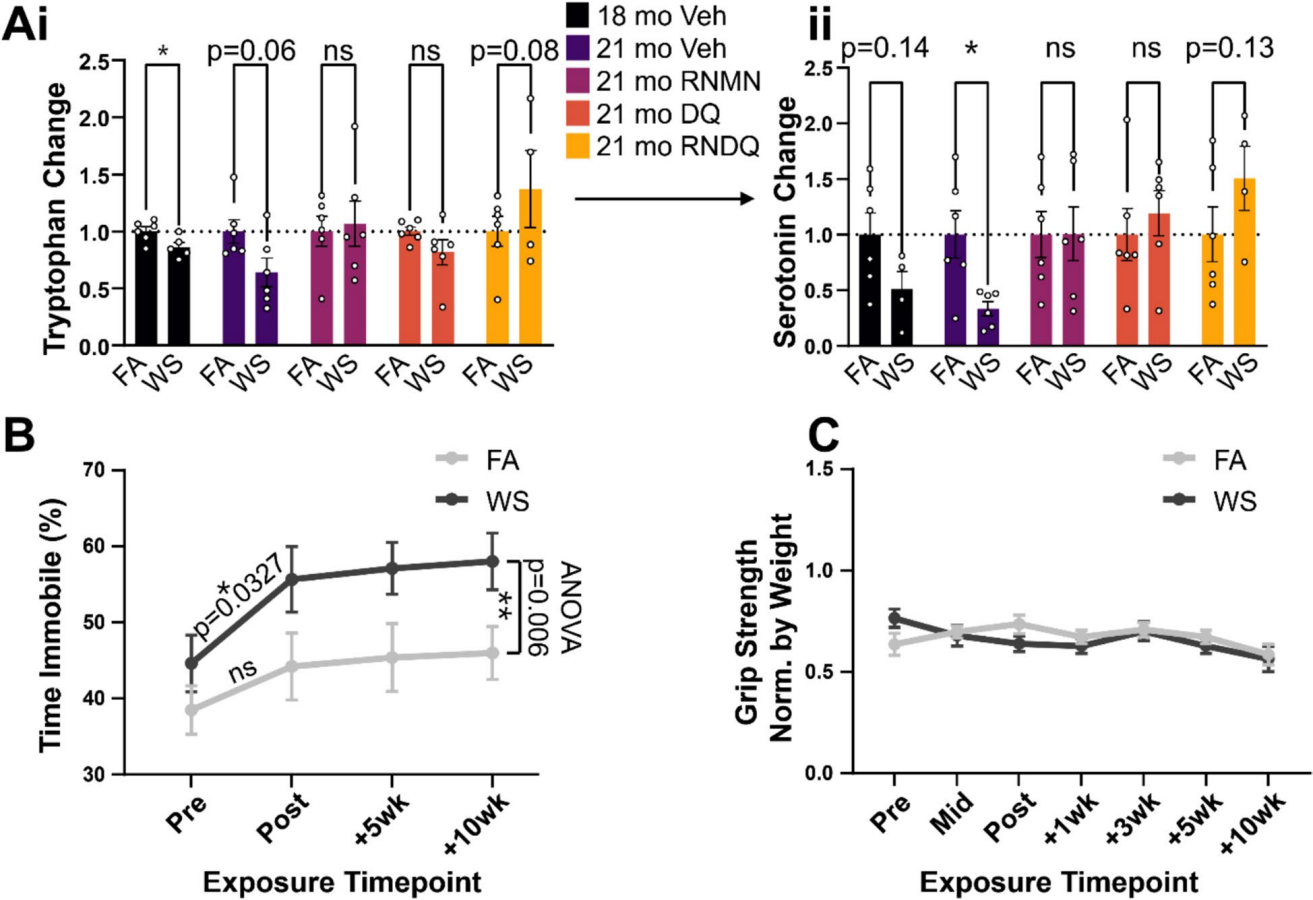
Metabolites and forced swim test measurements. **A** i & ii) Metabolites detected in the serotonin synthesis pathway. i) Upstream tryptophan. ii) Serotonin abundance. **B** Forced swim test. Two-way ANOVA test comparison for FA vs WS over time was conducted. **C** Grip. Asterisks denote significant difference from control (*p < 0.05; **p < 0.01)

We observed significantly reduced tryptophan, the serotonin precursor (Fig. 4Ai), immediately following the subacute WS exposure, and a trend toward persistent reduction after 10 weeks of intervention-free recovery (*p* = 0.06). These effects were even more prominent in serotonin levels, with a trending reduction immediately post-exposure (*p* = 0.14) and a significant decrease 10 weeks later (Fig. 4Aii). These data indicate a long-term reduction in serotonin that persists up to 10 weeks post-exposure in our model.

### Validation and further assessment of persistent neurological effects

To assess whether the persistent neurometabolomic alterations observed in the PFC of WS-exposed mice coincided with behavioral alterations and neuroinflammation, an additional group of female, 18-month-old mice was exposed to either FA (*n* = 10) or WS (*n* = 10) using a similar exposure paradigm. We then assessed the mice for behavioral changes for 10 weeks following exposures using a forced swim test and evaluated the mice for potential WS exposure induced neuroinflammation.

First, to examine the effect of WS-induced reductions in the PFC levels of serotonin on behavior, we performed forced swim tests 1 day before exposures, 1 day after the last exposure, and then 5 weeks and 10 weeks after the exposures (Fig. 4B). When examining the pre-exposure timepoint, WS-exposed mice showed no significant differences in mobility when compared to FA mice. However, in all post-exposure cases — immediately after exposure, + 5 weeks, and + 10 weeks — significant reductions in time spent mobile were observed in the WS-exposed group compared to FA controls. To validate that the reduced mobility observed in animals was a behavioral effect of the WS exposure and not caused by neuromuscular impairment, animals were subjected to a grip strength test. We observed no difference in grip strength between the FA control sand the WS-exposed mice, illustrating that these observed effects were not the result of sarcopenia (Fig. 4C). Taken together, these data support our finding of WS-induced alterations in PFC serotonin levels and indicate that inhalation of WS results in depression-like behavior that persists long after cessation of exposure.

**Fig. 5:**
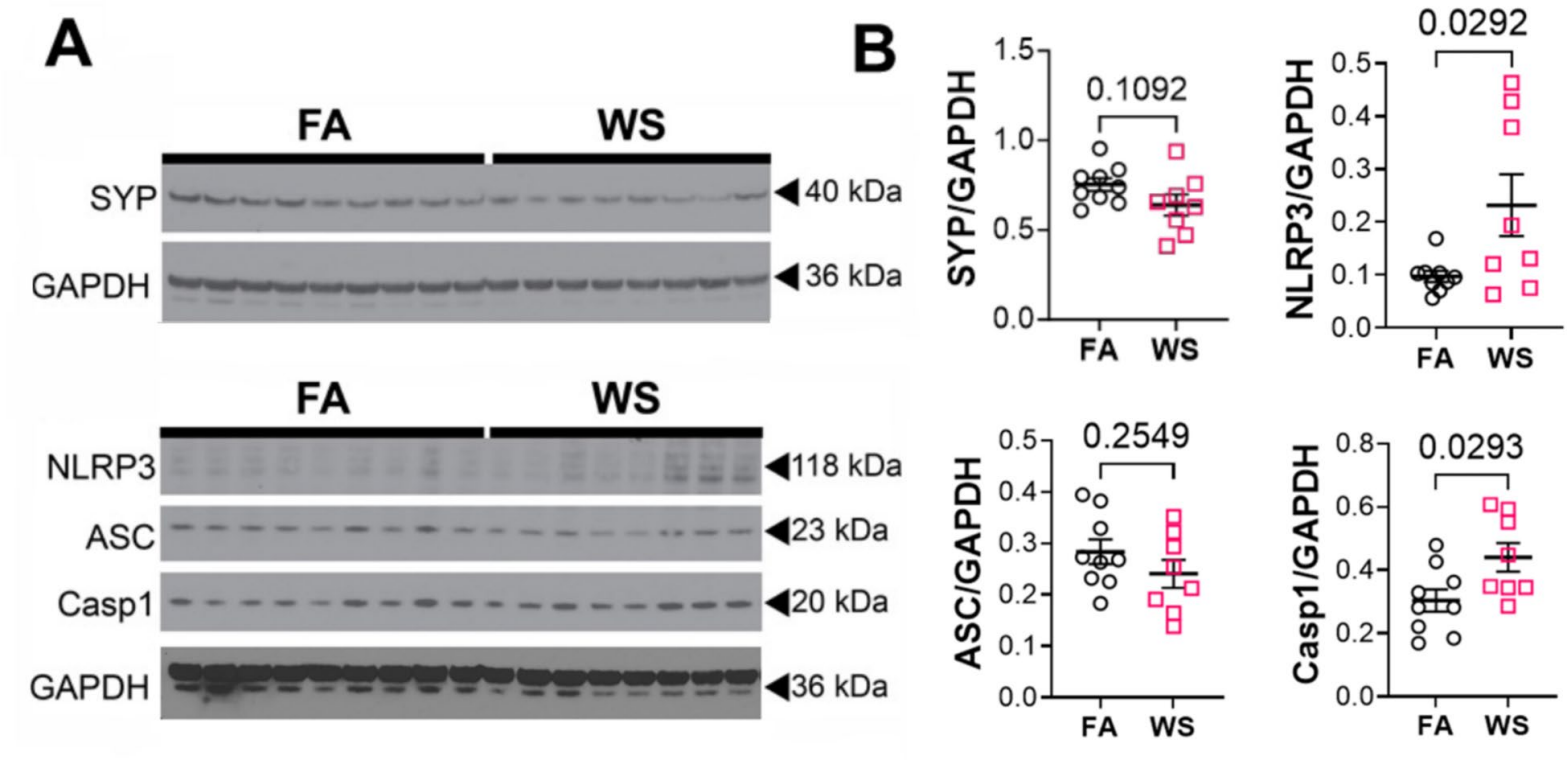
Sustained protein changes 70-days following exposure. **A** Western blot of hippocampal lysates for synaptophysin, NLRP3, ASC, and Casp1. **B** Quantifications of Western blot densitometry (comparison based on Student’s t-test)

To determine if exposure to WS induced impairment to synaptic structures, western blot analysis of hippocampal lysates was performed by immunoblotting for synaptophysin. A modest but not statistically significant reduction in synaptophysin levels was observed in the WS-exposed group compared to FA controls at 10 weeks post-exposure (Fig. 5A). Western blot analysis of hippocampal lysates was also conducted to assess persistent changes in nucleotide-binding oligomerization domain, leucine-rich repeat and pyrin domain-containing protein 3 (NLRP3) inflammasome levels, key neuroinflammatory proteins associated with long-term neurodegenerative disease [58–60]. 10 weeks after exposures, NLRP3 and caspase-1 (Casp1) levels were both significantly elevated in WS-exposed mice compared to FA controls, while apoptosis-associated speck-like protein containing a caspase-activation and recruitment domain (ASC) was unaltered (Fig. 5B). Multiplex ELISA using a pro-inflammatory panel of 10 cytokines was performed to assess persistent changes in mature cytokine levels. Of the cytokines assessed (IFN-γ, IL-1β, IL-2, IL-4, IL-5, IL-6, IL-10, IL-12, KC/GRO, and TNFα), none were significantly altered in WS-exposed mice compared to control at 10 weeks (Supplemental Fig. 5). Taken together, these data indicate that there is persistent elevation in inflammasome priming, but lack of overt cytokine maturation and release in the brain at 10 weeks post-exposure. Such persistent priming of the inflammasome may predispose the animals to increased reactivity to inflammatory stimuli, although further studies are needed to investigate this.

### Metabolomic similarities between WS exposure and aging

Finally, a comparison of metabolomic effects between aging and WS exposure was performed (Supplemental Fig. 6 A). A total of 129 significantly altered pathways were subset into those related to aging [51], with everything else listed as general (78, Supplemental Fig. 6B). From these data, there was a 70.5% overlap between aging and WS exposure (91 pathways, both general and age-related). A closer investigation of this subset revealed that 53 general pathways were shared between aging and WS exposure, and 38 age-related pathways were shared. The effect of aging perturbed 20 general pathways, and 5 general pathways were unique to WS exposure. Similarly, the non-exposed aging mice exhibited unique 9 additional age-related pathways. However, the exposure group exhibited a perturbation of 4 age-related unique pathways. Overall, our data show that the effects of 10 weeks of aging in the mouse brain resulted in a larger shift in the metabolic profile of the PFC than exposure to WS, both in terms of age-related and general pathways. However, although modest, the pathways altered in the PFC by WS exposure were similar to the pathways perturbed through aging.

Alteration of two key pathways were observed for both WS exposure and aging that aligned with the sustained behavioral and protein marker changes, namely “long-term depression” and “longevity”. The proteins/genes associated with long-term depression pathways impacted by WS exposure included GLRA4, LDHA, EPRS, MLNR, F2RL3, NGF, SLC29A3, PEMT, LCT, and ALDH2, while those impacted by aging included GLRA4, LDHA, EPRS, P2RY10, FFAR1, NGF, NTHL1, ADORA2B, VIP, CDS1, CKM, PRDM2. In the longevity regulating pathway, WS exposure affected proteins/genes included MPO, IL1B, CAD, GLUD2, FFAR1, GRIK5, SLC1A4, GOT1, BDKRB1, MLNR, KISS1R, and F2RL3, while aging affected MPO, IL1B, CAD, GLUD2, FFAR1, UROD, EEF1E1, AKT1, GNRH1, P2RY10, PROKR2, FFAR2, and ADORA2B. Supplemental Fig. 6 C illustrates the overlap in the proteins/genes mapped to these pathways.

## Discussion

This study examined the immediate and long-term neurometabolomic and neuroinflammatory effects after subacute WS exposure in a murine model of advanced aging, which revealed sustained reductions in PFC serotonin, elevated NLRP3 and Casp1 levels, and lasting behavioral changes up to 10 weeks after cessation of exposure. While the neurometabolomic results largely concur with recent studies in younger, 3-month-old animal models [17], the findings in older 18-month-old animals were more robust and persistent. Importantly, however, resveratrol + NMN treatment was most the beneficial intervention after WS exposure compared to exposure-matched, untreated controls. These findings suggest that there are nutritional/dietary opportunities to offset the detrimental impacts of exposure to wildfire smoke in advanced age, as an actionable public health approach. This is of particular importance as concern for the impacts of exposure to toxicants from wildfires increases globally.

The concentrations of WS used in the present study are similar to concentrations seen in earlier studies of naturally-occurring wildfire PM [16, 17], and well within range of human exposures that occur routinely during summer months [62]. Thus, we were specifically modeling an exposure concentration that millions of individuals may experience within a few hundred kilometers of the wildfire source, but sufficiently distant that evacuation would not be considered. Notably, our laboratory-based exposure system does not recapitulate the atmospheric aging associated with long-range transport, but the neuroinflammatory outcomes were quite similar between laboratory-generated and naturally occurring smoke exposures [16, 17]. Although many of the toxic gaseous components dissipate within close proximity to the fire source, carbon monoxide and PM_2.5_ can be transported over great distances in the air due to their stability. A recent study from our lab reveals PM_2.5_ from wildfire smoke drives neurological outcomes thousands of kilometers away from the fire origins [16].

The study was designed to investigate how WS exposure might alter the trajectory of natural aging, utilizing a window from 18 to 21 months of age when many physiological systems are in decline in mice. The selection of animal age was largely based on the increased susceptibility of older animals to environmental insults and exposures [63, 64]. Previously, data from our lab showed that 3-month-old female mice were unable to completely resolve WS exposure effects within 30 days of final exposure [17]. In the present study, we opted for a model of enhanced aging at 18–21 months of age to see whether this neuroinflammatory insult would prime a more rapid decline.

Human studies show a decreased ability to focus and learn after wildfire smoke exposure [15]. Moreover, numerous epidemiological studies support a link between PM and neurological and behavioral health [6, 65, 66], but few have addressed wildfire smoke, specifically. Neurological and psychiatric outcomes associated with PM_2.5_ exposure include depression, anxiety, suicide, ADRD, and decreased learning ability, in addition to cardiopulmonary outcomes that can be fatal [6, 11–13, 15, 67–69]. A recent study, however, observed that risk of dementia from wildfire-derived PM was positive along with dusts from agricultural sources, while most other sources of PM were not associated with dementia outcomes [14]. In our study, WS exposure caused a sustained reduction in serotonin levels in the PFC of aged mice that persisted even after 10 weeks of recovery. Decreases in serotonin in the PFC have been linked to depression-like behavior in both human and animal studies. In support of this evidence, mice exposed to WS exhibited increased immobility in the forced swim test that was not due to sarcopenia per the grip test. However, it is important to note that cardiorespiratory deficits may be an important outcome of WS exposure and could contribute to the observed.

Based on our experimental design, we were able to assess the neurobiological effects of WS exposure and natural aging. Previous work from our lab [16] and others [15, 70, 71] revealed alterations to metabolic profiles that were suggestive of mood alterations and impaired memory formation following WS exposure. Thus, we dissected the PFC to determine these effects and modeled them against aging, natural resolution of response, and the comparison of each pharmaceutical intervention. To further assist in determining age-related effects, we employed a metabolomic panel designed to investigate the NAD^+^ synthesis pathway, and an additional untargeted panel. This experimental design was specifically chosen because it afforded the ability to examine a multitude of effects. Namely, the immediate exposure effects were determined through a comparison of FA vs WS exposure 1-day post final exposure while the longer-term effects of WS exposure were evaluated with and without intervention through a comparison of the FA and WS-exposed mice at 10 weeks post-exposure.

While the metabolic shift observed in the PFC of mice exposed to WS persisted over 10 weeks, assessment of inflammatory cytokine levels measured in the hippocampus of these mice indicated that neuroinflammation was largely resolved after 10 weeks post-exposure. However, NLRP3 inflammasome protein levels remained significantly elevated indicative of persistent inflammasome priming in WS-exposed mice [72]. The inflammasome plays an important role in inflammaging, a chronic sterile inflammatory process in aging that contributes to increased morbidity and mortality [73–75]. Our findings suggest that subacute WS exposure may serve as an environmental trigger mediating the inflammaging process. Using our unique exposure paradigm, we were able to compare and contrast a multitude of neurometabolomic parameters, such as natural aging, WS-associated aging, and the potential for pharmaceutical intervention to mitigate the effects of WS. For example, the difference between natural aging can be examined by comparing FA 18-month-old vehicle treated mice to FA 21-month-old vehicle-treated mice. These naturally altered age-associated metabolites can be compared to those found in WS-exposed 18-month-old vehicle-treated mice vs WS-exposed 21-month-old vehicle-treated mice for overlap. Additionally, we cannot rule out the possibility of epigenetic alterations following exposure. The rate of epigenetic aging (biological age) has been measured against the chronological age as a marker of accelerated or decelerated aging. Although not investigated in this study, it would be worthwhile to determine the effects of wildfires and WS on the rate of neurological aging as this could shed light on the current state of unknown health risks.

### Limitations and summary

Several limitations for the study should be noted. As resveratrol activates NAD^+^ consuming enzymes for longevity-associated effects and NMN is a precursor to the enzymatic fuel source, NAD^+^, this combination was viewed strategically for the aging mouse model, but we did not assess the specific impact of either resveratrol or NMN in isolation. The choice of biomass (pinon wood) for this study was based on local fuel sources and may differ from the numerous other sources. Importantly, wildfires are uncontrolled mixtures of grasses, shrubs, and trees, burning at different temperatures, with meteorological factors influencing transport, mixing, and atmospheric aging of PM. Thus, there may not be a perfect model for wildfire smoke exposure, given the variations of time, distance, and concentration to which millions of people are exposed. We specifically examined the PFC for metabolomic data and made no comparisons to other regions of the brain. While we anticipate that neuroinflammation from WS exposures is widespread in the brain, different regions may respond differently than what was observed in the PFC. Similarly, we specifically examined the hippocampus for inflammasome proteins, inflammatory cytokines, and synaptophysin levels but did not assess other brain regions. Additionally, inflammatory cytokine and inflammasome levels were only assessed 10 weeks post-exposure to assess any persistent changes. We were unable to assess whether neuroinflammation increases acutely after WS exposure in this aged model, although, we have previously reported invasion of peripheral immune cells and reactive microglia within days to weeks after WS exposure in young 8-week-old mice [17]. Lastly, the current study only examined female mice and is not generalizable to both sexes of mice without investigation.

Summarily, these data reveal persistent alterations in the neurometabolome of aging mice following exposure to WS, with corroborating behavioral impacts and persistent inflammasome priming. The WS exposure tended to promote or exacerbate the metabolomic changes associated with aging, suggesting that not only is aging a risk factor that promotes vulnerability to environmental contaminants, but that environmental contaminants may drive certain processes of aging. Findings help explain the epidemiologically observed associations between PM exposure and neurological outcomes and further highlight the need to better understand the neurological and psychological impacts that wildfire smoke may have on public health along with practical interventions to offset those effects.

## Materials and methods

### Animals and wood smoke exposures

Female C57BL/6J mice (The Jackson Laboratory) at 18 months of age were housed in AAALAC-approved facilities, on a 12 h light:dark (7AM:7PM) cycle and allowed to acclimate for 1 week prior to experiments. Depending on experimental condition, they were provided either standard chow diet or resveratrol chow (0.1% w/w, Teklad Envigo) and DI water or nicotinamide mononucleotide (NMN, Millipore Sigma) water (described below) ad libitum (Fig. 1A). A total of 60 mice were used, randomly and evenly divided into filtered air (FA) control and woodsmoke (WS) exposed groups; with 6 mice per reusable plastic animal case system (Fig. 1A). All procedures were conducted humanely with approval by the University of New Mexico Institutional Animal Care and Use Committee. For exposures, a BioSpherix Medium A-Chamber was used, with mice in reusable shoebox plastic animal case systems. They were outfitted with standard wire tops, and water was available to mice throughout the exposures, but food was withheld (4 h/d).

Whole-body exposures to biomass combustion were conducted for 4 h every other day for 14 days (7 total exposures). Biomass smoke production was facilitated by a ceramic furnace encircling a quartz tube using chipped pinon wood as the fuel [17]. This tube was connected to a dilution chamber, with subsequent plumbing into the exposure chamber (Supplemental Fig. 1). Exposure chamber smoke abundance was facilitated by vacuum and/or pressurization, with total pressure monitoring to ensure min/max exposure chamber pressure never exceeded -/+ 25 mm Hg. Concentrations were monitored in real-time and manually adjusted to provide consistent average exposure levels.

Exposure concentrations were measured with a DustTrak II (TSI, Inc; Shoreview, Minnesota) in real-time using a tube fed directly into the exposure chamber. 47 mm quartz filters were collected for the duration of each exposure, and gravimetrically confirmed final daily averages were recorded using a microbalance (XPR6UD5, Mettler Toledo) in a temperature-controlled laboratory. Particle size distribution was quantified using the TSI Laser Aerosol Spectrometer 3340 A using a tube fed directly to the exposure chamber. Size distribution was acquired without mice in the exposure chamber, and distribution was measured over a single 2-h run. Particle size distribution largely fell within PM_1_ (median range = 0.138- 0.145 μm) with less than 1% of particles above PM_2.5_.

### Post-exposure recovery and pharmaceutical interventions

After the last round of exposures, one group of FA-exposed (*n* = 6) mice and one group of WS-exposed mice (*n* = 5) were euthanized. The remaining FA and WS-exposed mice were equally divided into 1 of the 4 intervention groups (vehicle control, RNNM, DQ or RNDQ) for 10 weeks after exposures (Fig. 1A). The intervention protocols were as follows: 1. Vehicle control intervention: standard chow, deionized (DI) water, gavage with vehicle; 2. RNMN intervention: resveratrol milled into standard chow, NMN added in DI water, gavage with vehicle; 3. DQ intervention: standard chow, DI water, DQ given via gavage; 4. RNDQ intervention: resveratrol milled into standard chow, NMN added in DI water, DQ given via gavage. An additional group of mice was included to recapitulate metabolomic findings of group 1 (vehicle control) in addition to applying a forced swim test and grip strength test to characterize potential behavioral implications. Resveratrol chow was achieved by milling 0.1% resveratrol by weight into standard chow (Envigo, WI, USA). Estimated intake of resveratrol was calculated as 5mg/30g mouse/day, or 167 mg/kg/day [76]. NMN was added to water 1.3mg/mL per week. Estimated intake of NMN was calculated as 300mg/kg/day [77]. Mice were gavaged with the formulation of Dasatinib (5 mg/kg, Thermo) and Quercetin (10 mg/kg, Thermo) in vehicle (60% Phosal, 10% ethanol, 30% PEG-400) or gavaged with vehicle alone; ethanol and PEG-400 Phosal formulation was 50% phosphatidyl choline and 50% propylene glycol (Thermo). Dasatinib and quercetin gavage stock was created the day of each administration. Drugs or vehicle were administered between 9:00 and 11:00 am for 3 consecutive days per week (i.e., Monday-Wednesday), every other week, for the total period of 10 weeks [78].

### Forced swim and grip strength testing

A separate subset of mice underwent exposures to FA (*n* = 10) or WS (*n* = 10) as above and then administered a forced swim test and grip strength test (Supplemental Fig. 2, Round 2). For the forced swim tests (conducted 1 day before and 1 day, 5 weeks, and 10 weeks after the subacute WS exposure) mice were placed into clear cylindrical plastic buckets measuring 24 cm in height and 19 cm in diameter, which were filled with 23–25°C tap water to a depth of 16–20 cm. Swim sessions were recorded over the course of a 6-min test, using a camera mounted on a tripod, which was positioned so its lens was level with the water surface in the bucket. For the pre-exposure test, an observer, unaware of the treatment conditions, analyzed the animals'behavior. A time sampling technique was employed to categorize the predominant behavior as swimming, immobility, or climbing. Additionally, behavior was scored automatically using DBscorerV2. Manual observations by the observer were scored every 5 s during the final 4 min of the test. Meanwhile, the DBscorerV2 scoring followed best practice guidelines provided in the original mauscript [79]. Briefly, videos were loaded, start and endpoints were selected, clean backgrounds were created without mice, regions of interest were marked, and auto-subject selection was performed. When video artifacts existed, background selection was cleaned, and delta area threshold was set at 2.6%. Comparisons between observer and DBscorer evaluations revealed no significant discrepancies. Consequently, DBscorerV2 was used for all subsequent swim tests.

Grip strength testing was performed using a force transducer (Series 2 Mark-10; JLW Instruments, USA) placed vertically measuring forelimb grip strength [80]. Peak tensions were measured between 8:00 and 11:00 am each testing day. Tests were performed in triplicate for each mouse with 1-min breaks between. Replicates were averaged and normalized by mouse weight taken on the same morning [81]. Procedurally, mice were allowed to grip the bar and the tail was slowly pulled, allowing mice to build up resistance to the force applied. In each replicate, trials resulting from a single paw grip, hind leg assistance, or a mouse body angle < 30° off center relative to the tensometer were excluded and repeated.

### Brain tissue dissection

Under isoflurane anesthesia, all mice underwent transcardial ice-cold 0.1M PBS (pH = 7.4) perfusion and subsequent steps were performed quickly. Surgical scissors were used to cut skulls from caudal to rostral along the medial edge until reaching the frontal bone anterior to bregma. Skulls were reflected rostrally to expose intact brains and olfactory bulbs. Brains were removed using a sterilized spatula and placed onto ice-cold watch glass with a sterile filter paper cover that had been soaked in ice-cold PBS (minus calcium and magnesium). Brains were hemi-sected with a sterile razor blade. Each left PFC was excised using a sterile razor blade and placed into a cryovial before plunging into liquid N_2_ for rapid freezing. Right hippocampi were micro-dissected using sterile fine forceps, wet-weights were recorded after placing into a cryovial, and then the tissue was rapidly frozen on dry ice and stored at −80°C for biochemical analysis.

### Tissue preparation for metabolomics analysis

Each PFC sample (~ 20 mg, *n* = 6) was homogenized in an Eppendorf tube using a Bullet Blender homogenizer (Next Advance, Averill Park, NY) in 200 μL methanol:PBS (4:1, v:v, containing 1,810.5 μM 13C3-lactate and 142 μM 13C5-glutamic acid) with a final addition of 800 μL methanol:PBS (same v:v). Samples were vortexed again for 10 s, stored at −20°C for 30 min and sonicated in an ice bath for 30 min. After a centrifugation step at 14,000 RPM for 10 min (4°C), 800 μL of supernatant was transferred to a new Eppendorf tube. The samples were dried under vacuum (CentriVap Concentrator, Labconco, Fort Scott, KS). The obtained residues were reconstituted in 150 μL 40% PBS/60% acetonitrile. A quality control sample was pooled from all the study samples.

### Liquid chromatography–tandem mass spectrometry (LC–MS/MS)

Targeted and untargeted LC–MS/MS techniques were similar to several recent reports [82, 83] using an Agilent 1290 UPLC-6490 QQQ-MS (Santa Clara, CA) system. Each PFC sample was injected twice: (1) 10 μL volume for negative ionization mode analysis and (2) 4 μL volume for positive ionization mode analysis. Both chromatographic separations were performed in hydrophilic interaction chromatography mode on a Waters XBridge BEH Amide column (150 × 2.1 mm, 2.5 μm particle size, Waters Corporation, Milford, MA). A flow rate of 0.3 mL/min, auto-sampler temperature of 4ºC, and a column compartment temperature of 40ºC were employed. The mobile phase was composed of Solvents A (10 mM ammonium acetate, 10 mM ammonium hydroxide in 95% H_2_O/5% acetonitrile) and B (10 mM ammonium acetate, 10 mM ammonium hydroxide in 95% acetonitrile/5% H_2_O). Initial 1 min isocratic elution of 90% B, decreased 40% B for 4 min (at t = 11 min till t = 15 min). The percentage of B gradually went back to 90%, to prepare for the next injection. The mass spectrometer is equipped with an electrospray ionization source. Untargeted data acquisition was performed in multiple-reaction-monitoring mode. A QC sample is pooled from all the study samples, and it is injected once every ten study samples to ensure instrument stability and data quality. The whole LC–MS system was controlled by Agilent Masshunter Workstation software (Santa Clara, CA). The extracted MRM peaks were integrated using Agilent MassHunter Quantitative Data Analysis (Santa Clara, CA). Metabolites were obtained by querying against 4 separate databases, including mzCloud, Metabolika, ChemSpider, and MassList. Final metabolites were selected based on a cutoff CV(QC) < 20% and an ID score of 1, where 1 is equivalent to a full match on predicted compositions, full match between all databases queried, and DDA for preferred ion. LC–MS grade acetonitrile, methanol, ammonium acetate, and acetic acid were purchased from Fisher Scientific (Pittsburgh, PA). Ammonium hydroxide was bought from Sigma-Aldrich (Saint Louis, MO). Standard compounds were purchased from Sigma-Aldrich and Fisher Scientific. Resultant data were normalized by tissue weight before subsequent normalization steps.

### Western blot analysis and ELISA

Each hippocampal sample (~ 25 mg, 10% weight/vol) was homogenized for 1 min on ice in an Eppendorf tube in Tissue Protein Extraction Reagent (TPER®, Thermo Scientific, Catalog #78,510) with phosphatase and protease inhibitor cocktails (Sigma Aldrich, P5726 and P8340, respectively) and sonicated with a probe-tip sonicator for 30 s at 20% A on ice. Soluble hippocampal lysates were centrifuged at 12,000 g for 30 min at 4 °C and aliquoted for ELISA or Western blot analysis.

Western blot samples were boiled for 15 min at 95 °C in NuPAGE™ LDS and RA (Invitrogen™, NP0007 and NP0004, respectively). Samples were resolved on NuPAGE™ 4–12% Bis–Tris gels (Invitrogen) and immunoblotted overnight on 0.45µM PVDF membranes (Thermo Scientific). Primary antibody dilutions were as follows: synaptophysin (Sigma, catalog # SAB4502906) 1:5000, NLRP3 (AdipoGen, Cryo-2) 1:2,000, ASC (AdipoGen AL177) 1:2500, caspase-1 (Santa Cruz, SC-398715) 1:1000, GAPDH (Millipore, AB2302) 1:10,000. HRP-conjugated secondary antibodies raised against their respective host species (goat-anti-mouse: Jackson ImmunoResearch, catalog # 115–035-003; goat-anti-rabbit: Jackson ImmunoResearch, catalog # 111–035-003) were consistently diluted at 1:10,000. Pierce ECL Western blotting substrate (Thermo Scientific, catalog # 32,109) was used to generate a chemiluminescent signal and immediately developed on radiography film. Scanned western blot films were analyzed using AlphaEaseFC™ v.3.2.1 and statistical analysis was performed using GraphPad Prism v.10.2.3.

Hippocampal lysates were also assessed for inflammatory cytokine levels using the Meso Scale Discovery© V-Plex Pro-Inflammatory Panel 1 Mouse Kit (MSD, K15048D-1). The assay was performed according to the manufacturer’s instructions using 50 µL of undiluted hippocampal lysates (10% w/v) in duplicate. The average concentration of inflammatory cytokine detected in each sample was then normalized to a total protein concentration of 100 µg for each sample determined by BCA assay (Thermo Scientific, catalog #23,225).

## Data analysis and statistics

For metabolomic analysis, the quality control samples (inserted at 10-sample intervals during mass spectrometry) were utilized as a pooled sample group to compensate for temporal variability of the instrument. Metabolomic data was analyzed using the R package MetaboAnalystR. Normality was determined via Shapiro–Wilk testing. In the event of normally distributed data, student’s t-tests were used. For non-normally distributed data, t-tests were employed on log_2_() or log_10_() transformed data. Tests were either conducted in GraphPad Prism v10.2.3, Excel (Version 2211 Build 16.0.15831.20098) or Rstudio v1.4.1564. Venn diagrams were generated using an online multiple list comparison tool [84]. For Venn diagrams, uncorrected student’s t-tests were performed as these data were not individually examined but qualitatively explored as overlapping matrices. These values were input into excel, Rstudio, or downloaded directly for figure generation. Volcano plots were generated using an FDR *p* < 0.1 and fold-change threshold > 1.5. Linear mixed effects regression modeling was performed with the packages limma, lme4, lmerTest, emmeans, multcomp, and lsmeans. Data were analyzed with fixed effects being age, exposure, and drug combination, with each metabolite being nested, and sample number called as a random effect. Results were holm-corrected before being plotted with the ggplot2, dplyr, and tidyr packages. Behavioral and other data were analyzed in GraphPad Prism using two-way ANOVAs or student’s t-tests.

Pathway analysis was performed using the metaboanalyst gene-metabolite network analysis tool. Statistically significant metabolites with fold-changes were aggregated from linear mixed effects regression (described above) and metabolites without direct match/hits within the tool were disregarded. Both the aging and exposure metabolites were input using a degree filter of 2.0, betweenness filter of 3.0, and minimum network was selected. The Metaboanalyst tool imputes the gene-metabolite interaction network to determine high-confidence gene pathways that may be altered. The exposure effect resulted in 1 subnetwork with 665 nodes, 2696 edges, and 62 seeds. The aging effects also resulted in 1 subnetwork, but with 731 nodes, 3303 edges, and 68 seeds. For each separate condition (aging or exposure), all nodes were used to determine statistically significant KEGG pathways and downloaded for further analysis. Pathways known to be associated with age-related alterations, depression, and those that have a direct effect on regulating longevity were determined through literature search via PubMed and ConsensusAI. When pathways overlapped between comparison conditions, they were color coded (e.g., longevity regulation pathway is statistically significant for aging and for exposure, color code in red). For numerical representation of overlapping pathways, UpSet plots were generated using the UpSetR package.

## Supplementary Information

Supplementary Material 1

## Data Availability

All data related to this study are publicly available upon reasonable request to the corresponding authors.

## Abbreviations

ADRD: Alzheimer's disease related dementias
ANOVA: Analysis of Variance
BCA: Bicinchoninic acid
D + Q: Dasatinib + quercitin
ELISA: Enzyme-linked immunosorbent assay
FA: Filtered air
NAD^+^: Nicotinamide adenine dinucleotide
NMN: Nicotinamide Mononucleotide
PM: Particulate matter
PFC: Pre-frontal cortex
RNMN: Resveratrol plus nicotinamide mononucleotide
WS: Woodsmoke
