# Supplementary material for: Neurometabolomic impacts of wood smoke and protective benefits of anti-aging therapeutics in aged female C57BL/6J mice
Source: Part Fibre Toxicol. 2025 Sep 1;22:23. doi: 10.1186/s12989-025-00639-4 (PMC12400709; doi:10.1186/s12989-025-00639-4)
Supplement: Supplementary file 1 — Supplementary Material 1 [file 12989_2025_639_MOESM1_ESM.docx]

**Data and Methods Supplement to:**

**NEUROMETABOLOMIC IMPACTS OF MODELED WILDFIRE SMOKE AND PROTECTIVE BENEFITS OF ANTI-AGING THERAPEUTICS IN AGED FEMALE C57BL/6J MICE**

**David Scieszka^1^, Jonathan Hulse^2^, Haiwei Gu^3^, Amanda Barkley-Levenson^1^, Ed Barr^1^, Marcus Garcia^1^, Jessica G Begay^1^, Guy Herbert^1^, Mark McCormick^4^, Jonathan Brigman^5^, Andrew Ottens^6^, Barry Bleske^7^, Kiran Bhaskar^2^, Matthew J Campen^1*^**

^1^Department of Pharmaceutical Sciences, University of New Mexico College of Pharmacy, Albuquerque, NM 87131

^2^Department of Molecular Genetics and Microbiology, School of Medicine, University of New Mexico Albuquerque, NM 87131

^3^Florida International University Center for Translational Sciences, Port St. Lucie, FL 34987

^4^Department of Biochemistry and Molecular Biology, School of Medicine, University of New Mexico Health Sciences Center, Albuquerque, New Mexico 87131

^5^Department of Neurosciences, Albuquerque, University of New Mexico School of Medicine, NM 87131

^6^Department of Anatomy and Neurobiology, Virginia Commonwealth University, PO Box 980709, Richmond, VA 23298

^7^Department of Pharmacy Practice and Administrative Sciences, University of New Mexico College of Pharmacy, Albuquerque, NM 87131

**Tables.**

**Supplemental Table 1. Pathways resulting from differential changes in metabolites (Figure 2).** Overlapping metabolites of shared Venn diagram region (196 metabolites) and WS-exclusive (162 metabolites).

| **Overlap** | | | | | | | |
| --- | --- | --- | --- | --- | --- | --- | --- |
| **Pathway** | **Total** | **Hits** | **Expected** | **Impact** | **Raw p** | **-log_10_(p)** | **FDR** |
| Alanine, aspartate and glutamate metabolism | 28 | 6 | 0.781 | 0.274 | 0.000 | 4.11 | 0.007 |
| Purine metabolism | 66 | 8 | 1.841 | 0.066 | 0.000 | 3.50 | 0.012 |
| Arginine biosynthesis | 14 | 4 | 0.390 | 0.732 | 0.000 | 3.37 | 0.012 |
| Pyrimidine metabolism | 39 | 5 | 1.088 | 0.118 | 0.004 | 2.42 | 0.079 |
| Nicotinate and nicotinamide metabolism | 15 | 3 | 0.418 | 0.478 | 0.007 | 2.14 | 0.099 |
| Histidine metabolism | 16 | 3 | 0.446 | 0.420 | 0.009 | 2.06 | 0.099 |
| Aminoacyl-tRNA biosynthesis | 48 | 5 | 1.339 | 0.078 | 0.009 | 2.03 | 0.099 |
| Nitrogen metabolism | 6 | 2 | 0.167 | 1.992 | 0.011 | 1.97 | 0.099 |
| D-Glutamine and D-glutamate metabolism | 6 | 2 | 0.167 | 1.992 | 0.011 | 1.97 | 0.099 |
| Pantothenate and CoA biosynthesis | 19 | 3 | 0.530 | 0.298 | 0.014 | 1.84 | 0.120 |
| Glycerophospholipid metabolism | 36 | 4 | 1.004 | 0.111 | 0.016 | 1.79 | 0.123 |
| beta-Alanine metabolism | 21 | 3 | 0.586 | 0.244 | 0.019 | 1.72 | 0.124 |
| Valine, leucine and isoleucine biosynthesis | 8 | 2 | 0.223 | 1.121 | 0.019 | 1.72 | 0.124 |
| Glutathione metabolism | 28 | 3 | 0.781 | 0.137 | 0.041 | 1.39 | 0.244 |
| Glyoxylate and dicarboxylate metabolism | 32 | 3 | 0.892 | 0.105 | 0.057 | 1.24 | 0.320 |
| Butanoate metabolism | 15 | 2 | 0.418 | 0.319 | 0.063 | 1.20 | 0.328 |
| Glycine, serine and threonine metabolism | 34 | 3 | 0.948 | 0.093 | 0.066 | 1.18 | 0.328 |
| Arginine and proline metabolism | 38 | 3 | 1.060 | 0.074 | 0.087 | 1.06 | 0.404 |
| Ether lipid metabolism | 20 | 2 | 0.558 | 0.179 | 0.105 | 0.98 | 0.465 |
| Taurine and hypotaurine metabolism | 8 | 1 | 0.223 | 0.560 | 0.203 | 0.69 | 0.852 |
| One carbon pool by folate | 9 | 1 | 0.251 | 0.443 | 0.225 | 0.65 | 0.860 |
| Vitamin B6 metabolism | 9 | 1 | 0.251 | 0.443 | 0.225 | 0.65 | 0.860 |
| Ascorbate and aldarate metabolism | 10 | 1 | 0.279 | 0.359 | 0.247 | 0.61 | 0.892 |
| Biosynthesis of unsaturated fatty acids | 36 | 2 | 1.004 | 0.055 | 0.266 | 0.58 | 0.892 |
| Arachidonic acid metabolism | 36 | 2 | 1.004 | 0.055 | 0.266 | 0.58 | 0.892 |
| Glycerolipid metabolism | 16 | 1 | 0.446 | 0.140 | 0.365 | 0.44 | 1.000 |
| Fructose and mannose metabolism | 18 | 1 | 0.502 | 0.111 | 0.401 | 0.40 | 1.000 |
| Citrate cycle (TCA cycle) | 20 | 1 | 0.558 | 0.090 | 0.434 | 0.36 | 1.000 |
| Pyruvate metabolism | 22 | 1 | 0.614 | 0.074 | 0.466 | 0.33 | 1.000 |
| Glycolysis / Gluconeogenesis | 26 | 1 | 0.725 | 0.053 | 0.524 | 0.28 | 1.000 |
| Folate biosynthesis | 27 | 1 | 0.753 | 0.049 | 0.537 | 0.27 | 1.000 |
| Galactose metabolism | 27 | 1 | 0.753 | 0.049 | 0.537 | 0.27 | 1.000 |
| Metabolism of xenobiotics by cytochrome P450 | 64 | 2 | 1.785 | 0.018 | 0.541 | 0.27 | 1.000 |
| Inositol phosphate metabolism | 30 | 1 | 0.837 | 0.040 | 0.576 | 0.24 | 1.000 |
| Porphyrin and chlorophyll metabolism | 30 | 1 | 0.837 | 0.040 | 0.576 | 0.24 | 1.000 |
| Cysteine and methionine metabolism | 33 | 1 | 0.920 | 0.033 | 0.611 | 0.21 | 1.000 |
| Amino sugar and nucleotide sugar metabolism | 37 | 1 | 1.032 | 0.026 | 0.653 | 0.18 | 1.000 |
| Valine, leucine and isoleucine degradation | 40 | 1 | 1.116 | 0.022 | 0.682 | 0.17 | 1.000 |
| Tyrosine metabolism | 42 | 1 | 1.171 | 0.020 | 0.700 | 0.15 | 1.000 |
| Primary bile acid biosynthesis | 46 | 1 | 1.283 | 0.017 | 0.733 | 0.13 | 1.000 |
|  |  |  |  |  |  |  |  |
| **WS Only** | | | | | | | |
| **Pathway** | **Total** | **Hits** | **Expected** | **Impact** | **Raw p** | **-log_10_(p)** | **FDR** |
| Aminoacyl-tRNA biosynthesis | 48 | 4 | 0.542 | 0.154 | 0.002 | 2.80 | 0.092 |
| Taurine and hypotaurine metabolism | 8 | 2 | 0.090 | 2.768 | 0.003 | 2.49 | 0.092 |
| Alanine, aspartate and glutamate metabolism | 28 | 3 | 0.316 | 0.339 | 0.003 | 2.48 | 0.092 |
| Arginine biosynthesis | 14 | 2 | 0.158 | 0.904 | 0.010 | 2.00 | 0.194 |
| Starch and sucrose metabolism | 15 | 2 | 0.169 | 0.787 | 0.012 | 1.94 | 0.194 |
| D-Glutamine and D-glutamate metabolism | 6 | 1 | 0.068 | 2.461 | 0.066 | 1.18 | 0.698 |
| Nitrogen metabolism | 6 | 1 | 0.068 | 2.461 | 0.066 | 1.18 | 0.698 |
| Arginine and proline metabolism | 38 | 2 | 0.429 | 0.123 | 0.066 | 1.18 | 0.698 |
| Valine, leucine and isoleucine biosynthesis | 8 | 1 | 0.090 | 1.384 | 0.087 | 1.06 | 0.812 |
| Phenylalanine metabolism | 12 | 1 | 0.135 | 0.615 | 0.128 | 0.89 | 1.000 |
| Glycerolipid metabolism | 16 | 1 | 0.181 | 0.346 | 0.167 | 0.78 | 1.000 |
| Pantothenate and CoA biosynthesis | 19 | 1 | 0.214 | 0.245 | 0.195 | 0.71 | 1.000 |
| Sphingolipid metabolism | 21 | 1 | 0.237 | 0.201 | 0.213 | 0.67 | 1.000 |
| Galactose metabolism | 27 | 1 | 0.305 | 0.122 | 0.266 | 0.58 | 1.000 |
| Glutathione metabolism | 28 | 1 | 0.316 | 0.113 | 0.274 | 0.56 | 1.000 |
| Glyoxylate and dicarboxylate metabolism | 32 | 1 | 0.361 | 0.087 | 0.307 | 0.51 | 1.000 |
| Cysteine and methionine metabolism | 33 | 1 | 0.373 | 0.081 | 0.315 | 0.50 | 1.000 |
| Glycine, serine and threonine metabolism | 34 | 1 | 0.384 | 0.077 | 0.323 | 0.49 | 1.000 |
| Glycerophospholipid metabolism | 36 | 1 | 0.406 | 0.068 | 0.339 | 0.47 | 1.000 |
| Pyrimidine metabolism | 39 | 1 | 0.440 | 0.058 | 0.361 | 0.44 | 1.000 |
| Valine, leucine and isoleucine degradation | 40 | 1 | 0.452 | 0.055 | 0.369 | 0.43 | 1.000 |
| Primary bile acid biosynthesis | 46 | 1 | 0.519 | 0.042 | 0.412 | 0.39 | 1.000 |
| Purine metabolism | 66 | 1 | 0.745 | 0.020 | 0.535 | 0.27 | 1.000 |

**Supplemental Figures:**

**A**

**
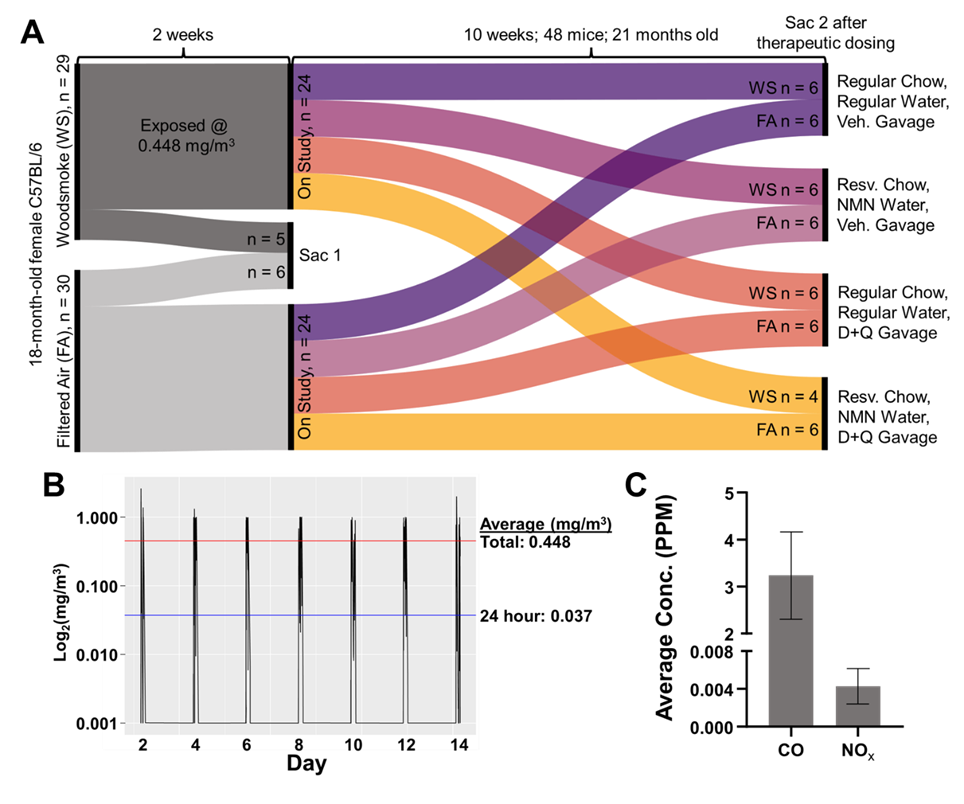
**

**
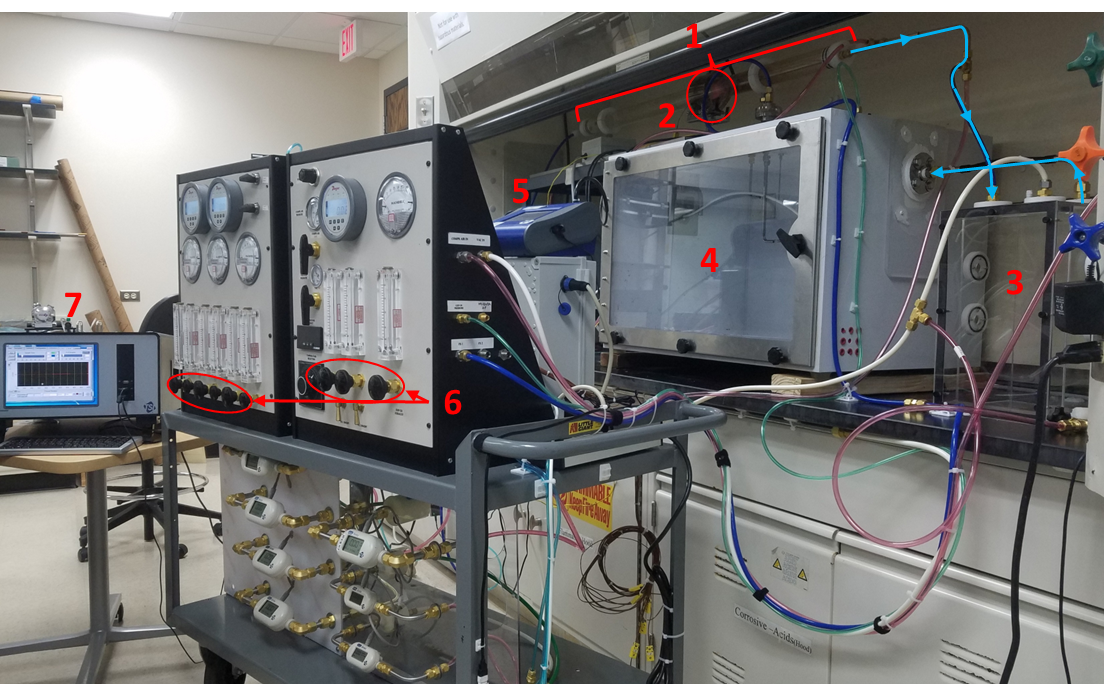
**

**Figure S1.** **Schematic of wood smoke system and exposure characterization**. A) Exposure chamber included: 1) Pre-weighed combustible material is placed inside of quartz boats that are lined inside of the quartz tube. 2) Furnace that runs along the length of the quartz tube; temperature can be altered in real time or set to a desired endpoint temperature. 3) Dilution chamber where smoke can enter via suction or pressurization from the quartz tube. 4) Exposure chamber that contained mouse cages; Smoke can enter via pressurization or vacuum suction. 5) Dust Trak was used to measure particle concentration in real time. 6) Banks of dials used to alter pressure and vacuum for increased or decreased smoke exposure. 7) TSI Laser Aerosol Spectrometer was used to measure particle size distribution. Arrows: direction of smoke flow. **B)** Concentration measuring during each 4-hour exposure. Top line: average across all exposures. Bottom line: average across all exposures, taking 24 hours per day into account. **C)** Levels of carbon monoxide (CO) and oxides of nitrogen (NOX). PPM: parts per million. Plotted are mean +/- SD.


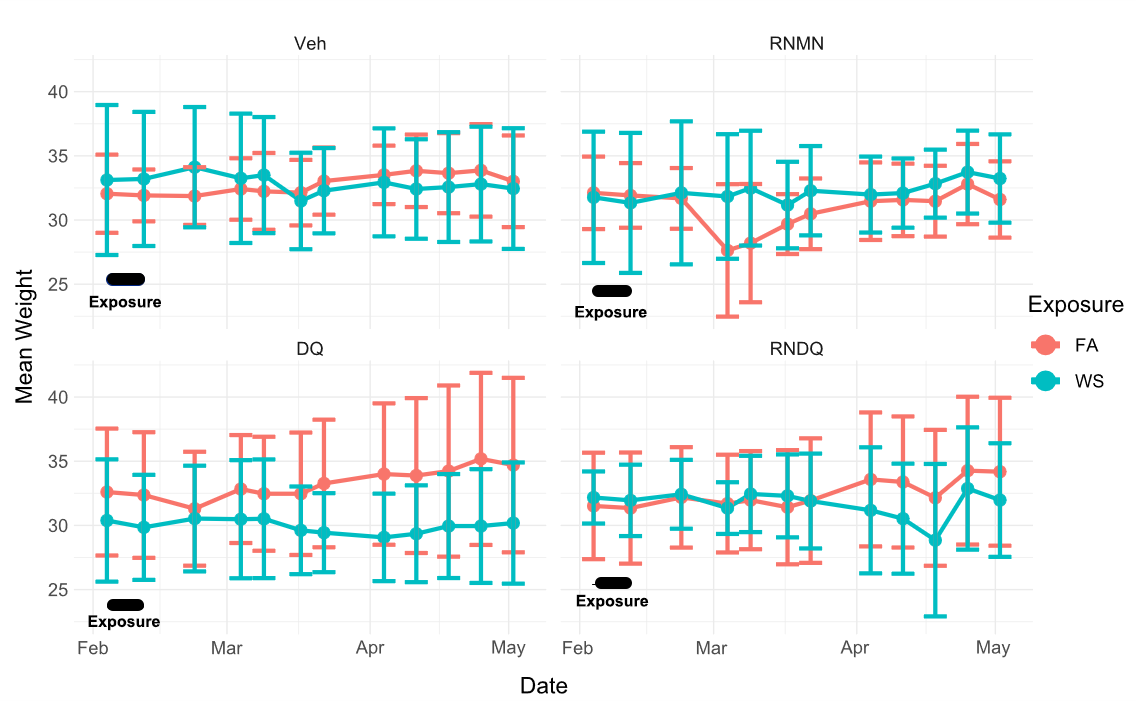


**Figure S2. Body weight trends across the exposure and intervention regimens.** No significant deviations from control groups were noted for any of the treatment groups. Mean +/- SD are shown.


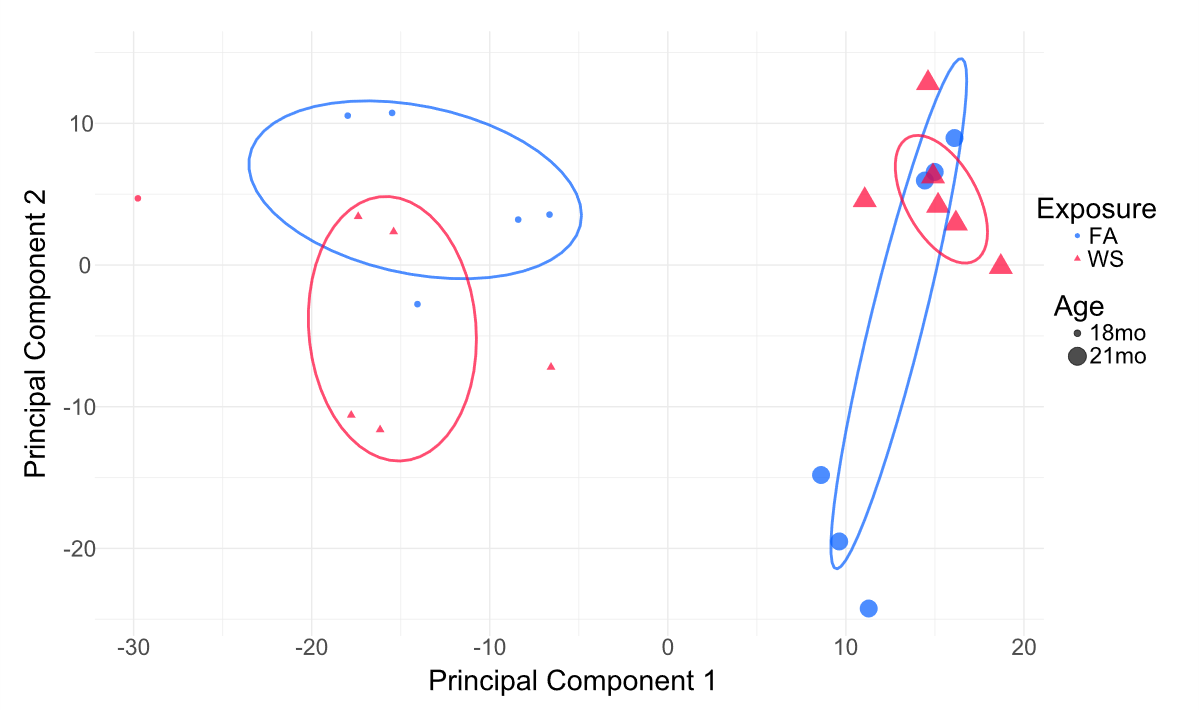

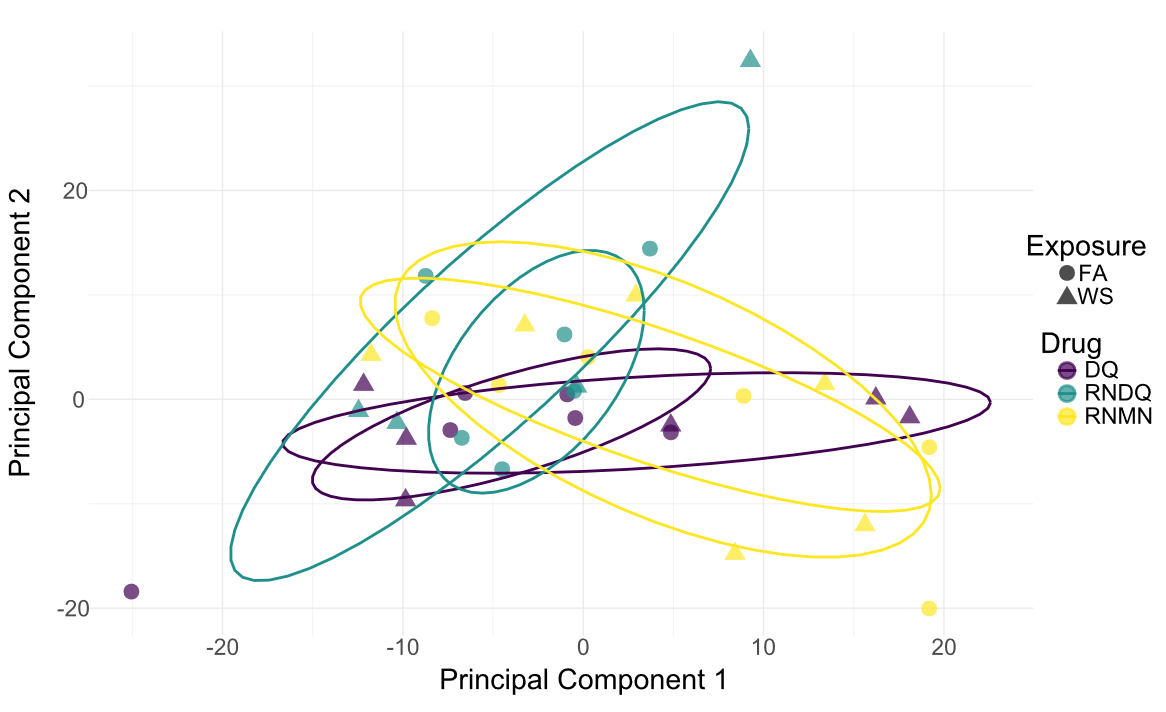


B.

A.

**Figure S3. Principal Component Analyses for PFC metabolomic data.** A. Comparing the effects of WS to FA in the 18 mo and 21 mo mice. Aging from 18 to 21 months represented a major overall neurometabolomic shift. B. Overlapping pathways for FA vs WS in each of the drug regimens.

**
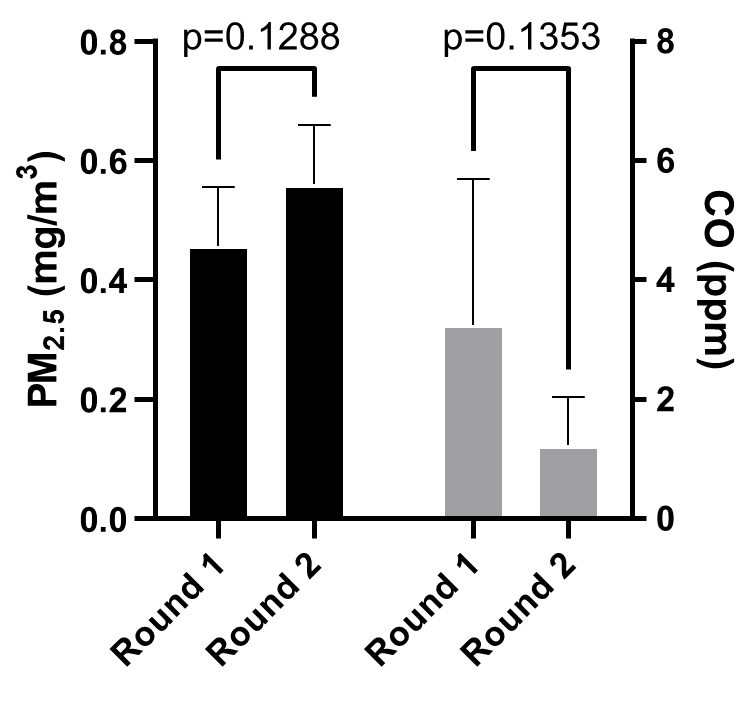
**

**Figure S4. Comparison of exposures between 1^st^ cohort and 2^nd^ cohort of animals used in forced swim tests.** Exposure paradigms shown as not significantly different between each other. Left axis: PM_2.5_ in mg/m^3^. Right axis: carbon monoxide in parts per million. Plotted are mean +/- SD.

**Figure S5. Inflammatory cytokine levels are not altered 70 days after exposure.** Multiplex ELISA panel of 10 inflammatory cytokines assessed in hippocampal lysates collected 10 weeks after exposure illustrating no sustained differences in neuroinflammation between filtered air control (FA; n=9) and wood smoke (WS) exposed mice (N=7). Student’s t-test was used to compare groups. Plotted are mean +/- SEM.


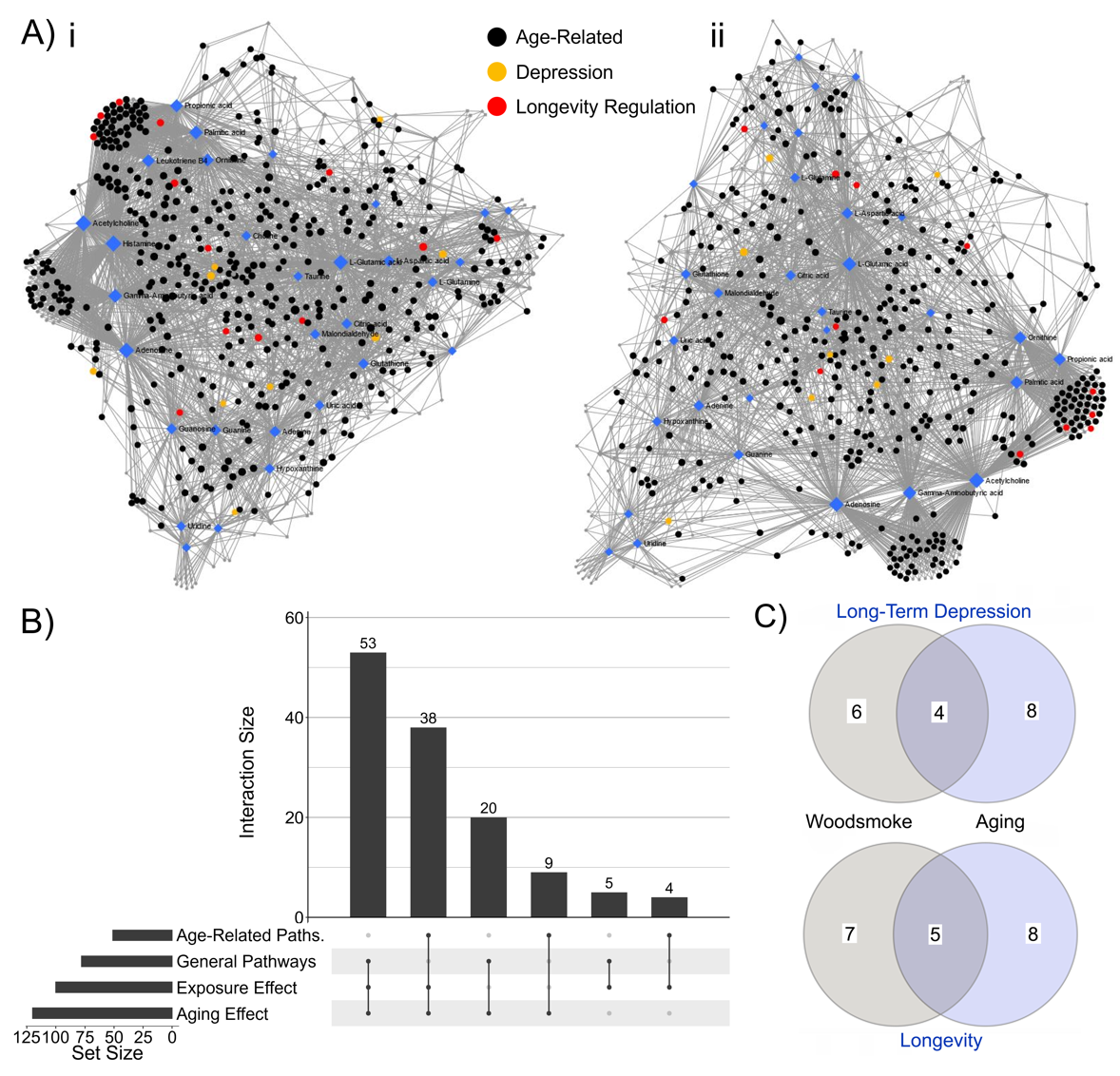


**Figure S6. Overlapping metabolite pathways between aging and wood smoke exposure from gene-metabolite pathway analysis.** **A)** Pathway analysis from statistically significant metabolites for each, Aging (i), and Exposure (ii) illustrating overlap between conditions based on age-related pathway alterations. Depression pathway and Longevity Regulation pathway were significantly altered in exposure and aging conditions. (colors: overlapping metabolites that were statistically significant; black: age-related pathways, yellow: depression pathway, red: longevity regulation pathway). **B)** Upset plot for numerical representation of overlapping and non-overlapping pathways from (A). Age-Related Pathway overlap (38) is lower than general pathway overlap between aging and exposure (53), illustrating similarities between aging and exposure that are not age-related. Exposure had 5 general pathways and 4 age-related pathways perturbed that were not observed in the aging condition. **C)** Venn diagrams showing overlap between Woodsmoke exposure and Aging pathway analysis for long-term depression (top) and Longevity (bottom). Long-term depression pathway metabolites that differed between by woodsmoke and filtered air groups included GLRA4, LDHA, EPRS, MLNR, F2RL3, NGF, SLC29A3, PEMT, LCT, and ALDH2. The long-term depression metabolites that were different in different age groups included GLRA4, LDHA, EPRS, P2RY10, FFAR1, NGF, NTHL1, ADORA2B, VIP, CDS1, CKM, PRDM2. The metabolites for longevity regulating pathway metabolites that were different between woodsmoke and filtered air groups included MPO, IL1B, CAD, GLUD2, GRIK5, SLC1A4, GOT1, BDKRB1, MLNR, KISS1R, FFAR1, F2RL3. The longevity regulating pathway metabolites that were different between ages included MPO, IL1B, CAD, GLUD2, UROD, EEF1E1, AKT1, GNRH1, P2RY10, PROKR2, FFAR2, FFAR1, ADORA2B.

**References**

1. Scieszka, D. *et al.* Biomass smoke inhalation promotes neuroinflammatory and metabolomic temporal changes in the hippocampus of female mice. *J Neuroinflammation* **20**, 192 (2023).

2. Bachmanov, A. A., Reed, D. R., Beauchamp, G. K. & Tordoff, M. G. Food Intake, Water Intake, and Drinking Spout Side Preference of 28 Mouse Strains. *Behav Genet* **32**, 435–443 (2002).

3. Mills, K. F. *et al.* Long-Term Administration of Nicotinamide Mononucleotide Mitigates Age-Associated Physiological Decline in Mice. *Cell Metabolism* **24**, 795–806 (2016).

4. Whole‐body senescent cell clearance alleviates age‐related brain inflammation and cognitive impairment in mice - Ogrodnik - 2021 - Aging Cell - Wiley Online Library. https://onlinelibrary.wiley.com/doi/10.1111/acel.13296.

5. DBscorer. (2023).

6. Takeshita, H. *et al.* Modified forelimb grip strength test detects aging-associated physiological decline in skeletal muscle function in male mice. *Sci Rep* **7**, 42323 (2017).

7. Justice, J. N. *et al.* Battery of behavioral tests in mice that models age-associated changes in human motor function. *AGE* **36**, 583–595 (2014).

8. Gu, H., Zhang, P., Zhu, J. & Raftery, D. Globally Optimized Targeted Mass Spectrometry: Reliable Metabolomics Analysis with Broad Coverage. *Anal. Chem.* **87**, 12355–12362 (2015).

9. Gu, H. *et al.* Quantitative Method to Investigate the Balance between Metabolism and Proteome Biomass: Starting from Glycine. *Angew Chem Int Ed Engl* **55**, 15646–15650 (2016).

10. COMPARE LISTS - MULTIPLE LIST COMPARATOR - Venn diagram generator, free online tool to find set intersections. https://molbiotools.com/listcompare.php.
